# CD4^+^CD25^+^FOXP3^+^ Treg Cells Induced by rSSP4 Derived from *T. cruzi* Amastigotes Increase Parasitemia in an Experimental Chagas Disease Model

**DOI:** 10.1155/2013/632436

**Published:** 2012-12-26

**Authors:** Y. Flores-García, J. L. Rosales-Encina, V. H. Rosales-García, A. R. Satoskar, P. Talamás-Rohana

**Affiliations:** ^1^Departamento de Infectómica y Patogénesis Molecular, Centro de Investigación y de Estudios Avanzados del Instituto Politécnico Nacional, 07360 Mexico, DF, Mexico; ^2^Unidad de Servicios Generales, Centro de Investigación y de Estudios Avanzados del Instituto Politécnico Nacional, 07360 Mexico City, DF, Mexico; ^3^Departments of Microbiology and Pathology, Medical Center, The Ohio State University, Colombus, OH 43210, USA

## Abstract

Currently, there is a considerable controversy over the participation of Treg cells during *Trypanosoma cruzi* infection, the main point being whether these cells play a negative or a positive role. In this work, we found that the adoptive transfer of CD4^+^CD25^+^FOXP3^+^ T cells from rSSP4- (a recombinant *Trypanosoma cruzi* amastigote derived protein, previously shown to have immunomodulatory properties on macrophages) immunized BALB/c donors into syngenic recipients simultaneously with *T. cruzi* challenge reduces cardiac inflammation and prolongs hosts' survival but increases blood parasitemia and parasite loads in the heart. These CD4^+^CD25^+^FOXP3^+^ Treg cells from immunized mice have a relatively TGF-**β**-dependent suppressive activity on CD4^+^ T cells. Therefore, regulatory CD4^+^CD25^+^ T cells play a positive role in the development of acute *T. cruzi* infection by inducing immunosuppressive activity that controls early cardiac inflammation during acute Chagas disease, prolonging survival, but at the same time promoting parasite growth.

## 1. Introduction

*Trypanosoma cruzi* is an intracellular protozoan parasite transmitted through the feces of blood-sucking insect vectors (*Triatoma*) and causes Chagas disease [1]. Intracellular amastigotes are responsible for the persistence of *T. cruzi* infection and induce inflammatory tissue damage in organs such as the heart, esophagus, and colon [2]. Currently, there is a considerable controversy over the participation of Treg cells during *Trypanosoma cruzi* infection, the main point being whether these cells play a negative or a positive role. Cytokines produced in response to infection with *T. cruzi* largely determine the immunopathology and susceptibility to disease. IL-10 and TGF-*β* both are differentiation factors of Treg cells. TGF-*β* production decreases elimination of parasites by macrophages (MΦs), associated with exacerbation of disease [3]. Similarly, IL-10 has also been associated with susceptibility to *T. cruzi* infection [4, 5] by blocking the production of IFN-*γ* by mouse spleen cells and inhibiting some IFN-*γ*-induced MΦ killing of intracellular *T. cruzi *[6, 7].

Parasites actively secrete or express molecules including parasite-derived proteins, lipids, and glycoconjugates that have potent effects on the immune system [8]. Newly transformed amastigotes, both intracellular and extracellular, express a major surface glycoprotein (SSP4) bound to the plasma membrane by a GPI anchor [9]. The gene that codifies for this protein was cloned [10], and rSSP4 was shown to be a modulator of the immune response, inducing high levels of IgG1, IgG2a and IgG2b isotypes, and the expression of iNOS and production of NO by MΦs [11]. Moreover, rSSP4 was also able to induce the mRNA for IL-1*α*, IL-6, IL-12, IFN-*γ*, and TNF-*α* cytokines in normal mice, and IL-10 in immunized mice [11], suggesting that TcSSP4 may be involved in modulating T cell populations during *T. cruzi* infection.

The goal of this study was to evaluate the role of antigen-specific induced CD4^+^CD25^+^ T cells during Chagas disease, either controlling or exacerbating infection by *T. cruzi*. Results show that indeed rSSP4 induced expansion of CD4^+^CD25^+^FOXP3^+^ T cells that exacerbate Chagas disease by promoting parasite proliferation during acute *T. cruzi *infection. These CD4^+^CD25^+^FOXP3^+^ Treg cells have a partially TGF-*β*-dependent suppressive activity on CD4^+^ T cells, indicating that these Treg cells play a positive role in the development of acute *T. cruzi* infection by inducing immunosuppressive activity.

## 2. Materials and Methods

### 2.1. Mice

Ten-week-old BALB/c mice from CICUAL (CINVESTAV, Mexico) were used. Mice were housed in a controlled microenvironment at the animal facility at CINVESTAV and managed according to institutional animal care guidelines.

### 2.2. Antibodies

Antibodies used in this work were APC-Cy7-anti-mouse CD4 (Cat. number 552051), purified anti-TGF-*β* (Cat. number 555052) and anti-IL-10, anti-TGF-*β* and anti-IFN-*γ* optEIA sets, from BD Bioscience (San Jose, CA, USA); APC-anti-mouse CD25 (Cat. number 17-0521), FITC or PE-anti-mouse FOXP3 (Cat. number 72-5775), PE-anti-mouse CD14 (Cat. number 12-0141), PE-anti-mouse CD19 (12-0193), from eBioscience (San Diego, CA, USA); purified anti-IL-10 (Cat. No. 505012) from Biolegend (San Diego, CA, USA).

### 2.3. Purification of Recombinant SSP4 (rSSP4)

*TcSSP4*, the gene that codifies for *T. cruzi* amastigote-specific surface antigen, was cloned in the EcoR1 site of the expression vector pMAL-C2, resulting in the plasmid pMAL-TcSSP4. *E. coli* DH5-*α* was transformed with this plasmid to obtain the fusion protein MBP::SSP4 (rSSP4) [10, 11]. rSSP4 and MBP were purified by amylase affinity chromatography; after purification, material was analyzed by 10% SDS-PAGE on which a 127 kDa protein corresponding to rSSP4 and a 43 kDa protein corresponding to MBP were observed, respectively (data not shown). In all experiments, purified MPB protein was included in restimulation conditions as a control, and no significant effects were observed with this protein; MBP alone did not induce CD4^+^CD25^+^FOXP3^+^ neither cytokines, as did the recombinant protein (data not shown).

### 2.4. Mice Immunization Protocol

Ten-week-old female BALB/c mice were divided into two groups, 3 mice per group. One group was treated with PBS (NIM), and the other one was immunized with rSSP4 protein (IM) once a week for 3 weeks (10 *μ*g per dose per mouse by intraperitoneal route). The number of repetitions of experiments is indicated in figure legends.

### 2.5. Flow Cytometry Analysis

Spleen cells from immunized and nonimmunized mice cultured for 72 h were stained, according to the desired cell markers, with one or more of the following antibodies: PE-anti-mouse CD19, PE-anti-mouse CD14, PE-anti-mouse CD4, APC-anti-mouse CD25, or FITC-anti-mouse FOXP3, according to the manufacture's protocol. In brief, nonspecific staining was blocked with anti-CD16/CD32 mAb (Fc block from eBioscience), and cells were incubated with the appropriate antibodies for 30 min on ice and washed twice with PBS containing 2% fetal bovine serum. For FOXP3 staining, cells were fixed/permeabilized for 45 min using a FOXP3 kit from eBioscience. Cells were then washed with 1X permeabilization buffer and stained with FITC-anti-FOXP3. Analysis of intracellular FOXP3 was performed according to the manufacturer's instructions. Briefly, spleenocytes were resuspended at 2 × 10^6^ cells/mL in complete medium or the conditions mentioned above for 72 h. Cells were stained with PE-Cy5.5-anti-CD4 and APC-anti-CD25, then cells were simultaneously fixed and permeabilized with Fix/Perm Buffer (eBioscience), and intracellular staining with PE-anti-FOXP3 mAb was developed. Cellular population analyses were performed with a FACS Calibur Becton Dickinson Cytometer (San Diego CA, USA) by acquiring 1 × 10^5^ events (gated by forward and side scatter properties; in the case of intracellular staining, these parameters were adjusted accordingly) and analyzed using Summit Software (Beckmann Coulter; Brea, CA, USA).

### 2.6. Isolation of CD4^+^ and CD4^+^CD25^+^ T Cells

Freshly isolated spleen CD4^+^ and CD4^+^CD25^+^ T cells from immunized mice were purified by positive selection by flow cell sorting. First, CD4^+^ and CD4^+^CD25^+^ T cells were stained with labeled antibodies APC-Cy7-anti-mouse CD4 and APC-anti-mouse CD25 for 30 min. Cell suspensions were passed through a high speed flow cytometer MoFlo from Beckman Coulter. Positively selected CD4^+^ and CD4^+^CD25^+^ cells were found to be more than 95% pure on FACS analysis.

### 2.7. *T. Cruzi* Challenge of Naïve, Immunized, and Adoptively Transferred Mice

CD4^+^CD25^+^ T cells were isolated from the spleens of naïve or rSSP4-immunized BALB/c mice as described previously. Nonprimed or rSSP4-primed CD4^+^CD25^+^ T cells (1 × 10^6^) were adoptively transferred into five-weeks-old female BALB/c mice through the tail vein. These mice were immediately infected with blood trypomastigotes of *Trypanosoma cruzi* (8 × 10^4^, H8 Yucatan strain), by intraperitoneal inoculation; naïve and immunized mice (3-4 animals) were infected following the same protocol. Parasitemia and survival rates were calculated. Naïve infected mice and nonprimed CD4^+^CD25^+^ T cells-transferred mice were used as controls.

### 2.8. Inflammatory Infiltrates and Amastigote Nests in the Cardiac Parenchyma

Areas of inflammation and nests of amastigotes were manually selected from photomicrographs, using the image software Image J (available at http://rsb.info.nih.gov/ij/index.html). Selected areas were quantified as pixels numbers, and determination of the relative area, corresponding to inflammation or amastigote nests, was obtained by dividing the area of interest into the total number of pixels and multiplied by 100. Analyses were performed in four different sections of the same heart.

### 2.9. T Cell Proliferation Assays

For *in vitro* proliferation, spleens from nonimmunized and immunized mice were excised aseptically 15 days after the third rSSP4 immunization, and cells were cultured in flat-bottom 24 − (2 × 10^6^) or 96 − (0.8 × 10^6^) well plates (Costar) in complete D-MEM medium for cytokine determination and proliferation, respectively. Cells were stimulated with Concanavalin A (ConA) (6 *μ*g/mL), MBP (5 *μ*g/mL), rSSP4 (10 *μ*g/mL), or in medium alone. Proliferation was measured after 72 h by [*methyl*-^3^H]TdR (Amersham) incorporation (1 *μ*Ci per well). Cells were harvested onto glass filters, placed in scintillation fluid, and counted in a Beckman scintillation counter. For cytokines measurements by ELISA, supernatants from 24 well plates were recovered, and to discard contamination with endotoxins, cells were induced to proliferate in the presence of Polymixin B (100 U/mL).

### 2.10. Suppression Assays

Suppression assays were performed as described by Gavin et al. [12]. Briefly, CD4^+^CD25^−^ T cells (5 × 10^4^), CD4^+^CD25^+^ T cells (titrating amounts) or a combination of the two populations were stimulated for 72 h with 1 × 10^5^ APCs (12 Gy irradiated spleen cells from nonimmunized mice). This was done in the presence of anti-CD3 (25 *μ*g/mL) plus anti-CD28 (2 *μ*g/mL) and rSSP4 (10 *μ*g/mL) in 96 well plates; in all conditions, cells were pulsed with 1 *μ*Ci/well of [*methyl*-^3^H]TdR (Amersham) for the final 16 h. Results are presented as mean±SD cpm values of triplicate wells. These experiments were also developed in the presence of anti-IL-10 (5 and 10 *μ*g/mL) or anti-TGF-*β* (5 *μ*g/mL) neutralizing antibodies. Viability of anti-IL-10 and anti-TGF-*β* antibodies was confirmed by western blot (data not shown).

### 2.11. Analysis of TGF-*β* mRNA Levels by RT-PCR

Total RNA was isolated using TRIzol reagent (Invitrogen) from spleen cells cultured in 24 well plates with different treatments for 24 h. RNA (5 *μ*g) was transcribed to cDNA with oligonucleotides (poly(dT)_16_), and SuperScript II reverse transcriptase, and PCR was performed with primers for TGF-*β* (sense; GCCCTGGATACCAACTATTGC, antisense; TCAGCTGCACTTGCAGGAGTAGCG) [13] and GAPDH sequences (as internal control; sense, CCTTCATTGACCTCAACTAC, antisense, GGAAGGCCATGCCAGTGAGA). Each PCR cycle consisted of a denaturation step (95°C, 1 min), an annealing step (65°C, 30 sec), and an elongation step (72°C, 30 sec). DNA was amplified for 30 cycles in a Bio-Rad Thermocycler. PCR products were analyzed on 1.5% agarose gel and stained with ethidium bromide.

### 2.12. Cytokines ELISA

IL-10, TGF-*β*, and IFN-*γ* were quantified by ELISA (BD optEIATM ELISA Kit) in culture supernatant of cells under different conditions of restimulation, as described above, according to the manufacture's protocol. Briefly, 96 well flat bottom plates were coated with capture antibody (dilution 1/250 for IL-10 and TGF-*β*, and 1/2,000 for IFN-*γ*), blocked with 10% PBS-FCS, washed three times, and incubated with the antigen for 2 h. After washing the plates, they were incubated with detection antibody coupled to avidin-HRP (horseradish peroxidase, 1/250 dilution); after several washes, substrate solution was added and the reaction was stopped after 30 min with 2 N H_2_SO_4_. Plates were read at 450 nm using a microplate reader (Bio-Rad model 680).

### 2.13. Statistical Analysis

Analyses were performed using GraphPad Prism version 5.0 software or Sigma Plot 10.0. Differences were considered statistically significant when a *P* value of less than 0.05 was obtained by Student's *t* or square Chi test.

## 3. Results

### 3.1. Mice Receiving rSSP4-Primed CD4^+^CD25^+^ T Cells but Not Mice Receiving Nonprimed CD4^+^CD25^+^ T Cells Show Exacerbation of Acute *T. cruzi *Infection

**Figure 1:**
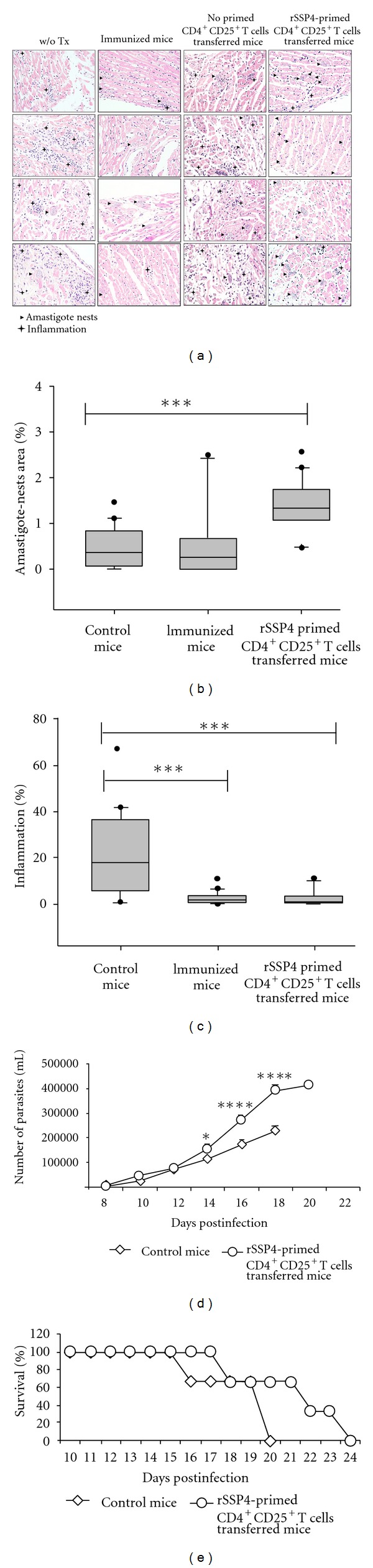
Mice receiving rSSP4-primed CD4^+^CD25^+^ T cells but not mice receiving nonprimed CD4^+^CD25^+^ Tcells show exacerbation of acute *T. cruzi *infection and prolonged survival rate. (a) Histological examination by hematoxylin-eosin staining of cardiac tissue from untreated (w/o Tx), rSSP4 immunized, nonprimed- or rSSP4-primed-CD4^+^CD25^+^ T cells-transferred mice. Amastigotes nests are shown by arrow heads and inflammation foci with +. Magnification 40x. (b) Amastigotes nests area between different groups is calculated using Image J program. Graphs represent the average measure of 20 fields. (c) Percentage of inflammation foci was calculated considering the whole area in 20 fields using J program. Statistical analysis was done using Sigma Plot 10.0. (d) Parasitemia levels measured by direct parasite counting from blood samples. (e) Survival rate of infected mice. For (d) and (e), results are the average of three independent experiments; statistical analysis was done with GraphPad Prism 5.0.

To evaluate the role of regulatory T cells and specifically rSSP4-induced CD4^+^CD25^+^FOXP3^+^ T cells during the acute phase of *T. cruzi* infection, CD4^+^CD25^+^ T cells (more than 96% pure) were purified from rSSP4-treated and from nontreated mice and transferred to naïve BALB/c mice just prior to *T. cruzi* challenge. *T. cruzi-*infected recipient mice receiving CD4^+^CD25^+^ T cells from rSSP4-immunized donors developed significantly less severe cardiac inflammation (+) (Figure 1(a), right panels; Figure 1(c)) but higher heart parasite loads (▸)(Figure 1(a), right panels; Figure 1(b)) and higher blood parasitemia (○) compared to controls (*◊*) (Figure 1(d)). Interestingly, mice receiving rSSP4-primed CD4^+^CD25^+^ T cells (○) also survived longer than controls (*◊*) (Figure 1(e)). On the contrary, when mice were transferred with CD4^+^CD25^+^ Treg cells from naïve mice, blood parasitemia showed the same level as control mice (data not shown). When cardiac tissue was examined for the presence of amastigote nests and inflammatory foci (Figure 1(a), third panel from left to right), they showed the same appearance as that seen with control mice (Figure 1(a), left panels), that is, there were few amastigote nests, always surrounded by inflammation, and, in general, inflammation was more accentuated in these two conditions. These results indicate that rSSP4-induced CD4^+^CD25^+^ T cells although control immunopathology, they promote parasite proliferation during acute *T. cruzi *infection. Moreover, rSSP4 immunized mice (Figure 1(a), second panels from left to right) showed a similar behavior after *T. cruzi* infection in terms of cardiac inflammation (+) and parasite load (▸) (Figures 1(a)–1(c)), as well as in survival rate and blood parasitemia (data not shown). Natural Treg cells present in naïve mice or natural Treg cells that came from nonimmunized mice and were adoptively transferred to naïve mice were unable to control inflammation and/or to promote parasite growth.

### 3.2. Spleen CD4^+^CD25^+^FOXP3^+^ T Cells Are Induced Upon Restimulation with rSSP4

**Figure 2:**
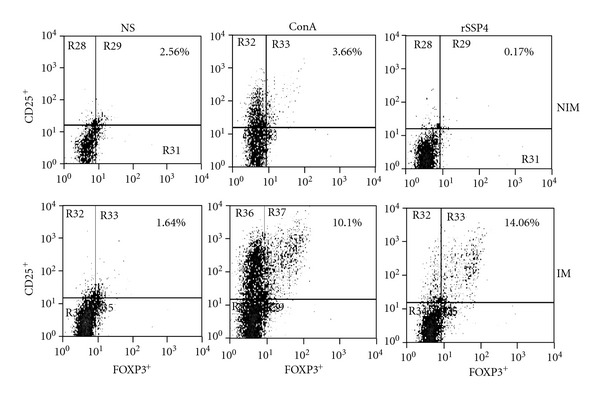
Spleen CD4^+^CD25^+^FOXP3^+^ T cells are rSSP4-specific. Mice were i.p. immunized with 10 *μ*g/mouse of rSSP4 or administered PBS. After three immunizations, spleen cells were stimulated *in vitro* with ConA, rSSP4, or nonstimulated for 72 h, and surface expression of CD25 and intracellular expression of FOXP3 were measured in spleen cells from both nonimmunized and immunized mice. Shown cells are CD4^+^ lymphocytes. Results are representative of four independent experiments.

Because we found that CD4^+^CD25^+^FOXP3^+^ T cells induced by immunization with rSSP4 promoted the development of Chagas disease, we wanted to see whether these regulatory T cells were antigen specific. Flow cytometric analysis revealed that rSSP4-stimulated spleen cells from rSSP4-immunized mice contained significantly higher percentage of CD4^+^CD25^+^FOXP3^+^ T cells as compared to similarly stimulated spleen cells from nonimmunized mice (14.06% and 0.17%, resp.) (Figure 2, right panels). No significant difference was noted in percentage of CD4^+^CD25^+^FOXP3^+^ regulatory T cells, in spleen cells from rSSP4-immunized or nonimmunized mice in the absence of stimulation (Figure 2, left panels), or in the presence of MBP (data not shown). These results indicate that rSSP4 promotes *in vitro* induction of FOXP3^+^ regulatory T cell population in an antigen-specific manner, because in the absence of stimuli or in the presence of MBP, which is part of the rSSP4, the proportion of T reg cells remained low.

### 3.3. rSSP4 Immunization Induced CD4^+^CD25^+^ T Cells with Suppressive Function *In Vitro*

**Figure 3:**
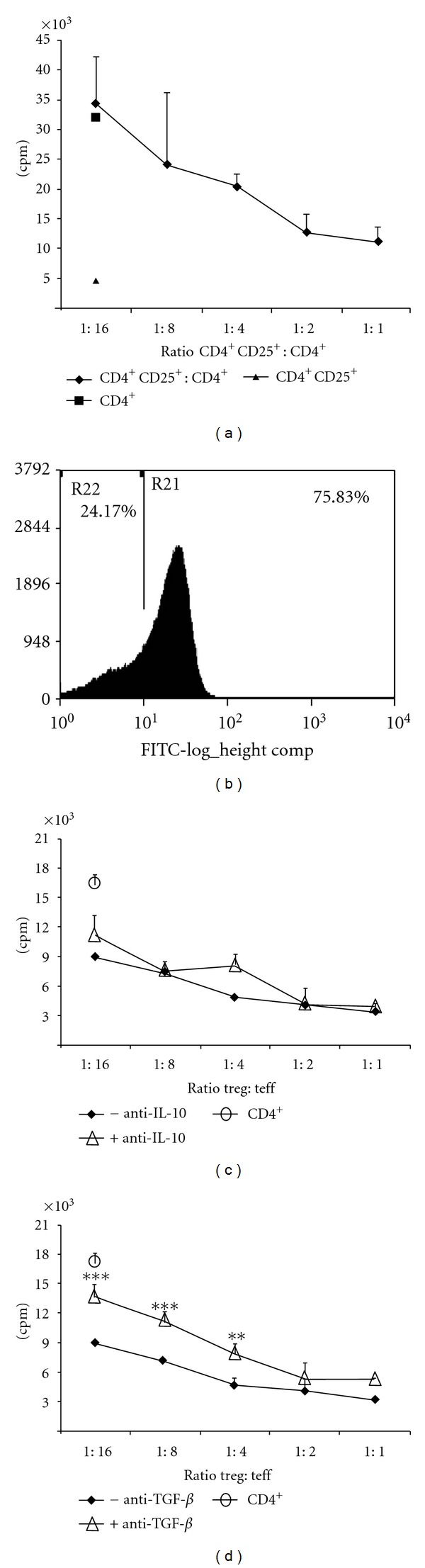
rSSP4 immunization induced CD4^+^CD25^+^ T cells with suppressive function *in vitro*. (a) A total of 5 × 10^4^ CD4^+^CD25^−^ T cells (from naïve mice) and 1 × 10^5^ APCs (from nonimmunized mice) were cultured alone or in combination with titer quantities of rSSP4-primed CD4^+^CD25^+^ T cells in the presence of anti-CD3 and anti-CD28 antibodies plus antigen. Proliferation was quantified by ^3^H-Thymidine incorporation during the last 16 h of culture. (b) An aliquot of rSSP4-primed CD4^+^CD25^+^ sorted T cells was tested for FOXP3 expression. Results shown are representative of three independent experiments (c) and (d). Anti-TGF-*β* antibodies partially inhibit suppressor activity of CD4^+^CD25^+^ T cells. A total of 5 × 10^4^ CD4^+^ T cells (from immunized mice) and 1 × 10^5^ APCs (from nonimmunized mice) were cultured alone or in combination with titer quantities of rSSP4-primed CD4^+^CD25^+^ T cells in the presence of anti-CD3 and anti-CD28 antibodies plus antigen in the presence of 5 *μ*g/mL of neutralizing anti-IL-10 (c) or anti-TGF-*β* (d) antibodies. Proliferation was quantified by ^3^H-Thymidine incorporation during the last 16 h of culture. Results are the average of three independent experiments done in duplicates.

A characteristic feature of regulatory T cells is their ability to inhibit cell proliferation of effector T cells, and once we evaluated the role of rSSP4-induced CD4^+^CD25^+^FOXP3^+^ T cells *in vivo*, we proceeded to perform suppression assays in order to confirm, *in vitro*, their suppressive capacity. CD4^+^CD25^+^ T cells induced after rSSP4 immunization exert a suppressor function on naïve CD4^+^CD25^−^ T cells. Freshly isolated spleen CD4^+^CD25^+^ T cells from immunized mice showed suppressive activity over *in vitro* activated CD4^+^ T cells, such that their activity increased with increasing numbers of Treg cells. Clearly a suppressive activity could be observed at the ratio of 1 : 4 (Treg : Teff), (Figure 3(a)). CD4^+^CD25^+^ T cells from nonimmunized mice showed a weaker suppressive activity (data not shown). CD4^+^CD25^+^ T cells used for suppressive assays were analyzed to determine whether they also express FOXP3^+^. Around 75% of these cells were positive for the presence of FOXP3 (Figure 3(b)). These results show that CD4^+^CD25^+^FOXP3^+^ T cells induced in rSSP4 immunized mice exhibit a strong suppressor activity.

### 3.4. Anti-TGF-*β* Antibodies Partially Inhibit Suppressor Activity of rSSP4-Primed CD4^+^CD25^+^ T Cells

In order to understand the mechanism of Treg cells suppression and confirm or deny the role of IL-10 and TGF-*β* as suppressor cytokines under these experimental conditions, we developed further experiments assessing the role of IL-10 and TGF-*β* and their suppressive function using anti-IL-10 or anti-TGF-*β* neutralizing antibodies as previously described [14]. Blockade of IL-10 using anti-IL-10 Ab (5 *μ*g/mL) had minimal or no effect on suppressive activity of rSSP4-primed CD4^+^CD25^+^ T cells (Figure 3(c)); a higher concentration of anti-IL-10 Ab (10 *μ*g/mL) did not affect either (data not shown). On the other hand, neutralization of TGF-*β* partially blocked suppressive activity of these cells as indicated by higher proliferation of T effector cells cocultured with rSSP4-primed CD4^+^ CD25^+^ T cells in the presence of anti-TGF-*β* antibodies (5 *μ*g/mL) indicating that anti-TGF-*β* restores CD4^+^ T effectors cells proliferation (Figure 3(d)). Taken together, these findings suggest that suppressor activity of rSSP4-induced CD4^+^ CD25^+^ T cells is TGF-*β* but not IL-10 dependent (Figures 3(c) and 3(d)).

### 3.5. Immunization with rSSP4 Induces TGF-*β* mRNA Expression and TGF-*β*, IL-10, and IFN-*γ* Production

**Figure 4:**
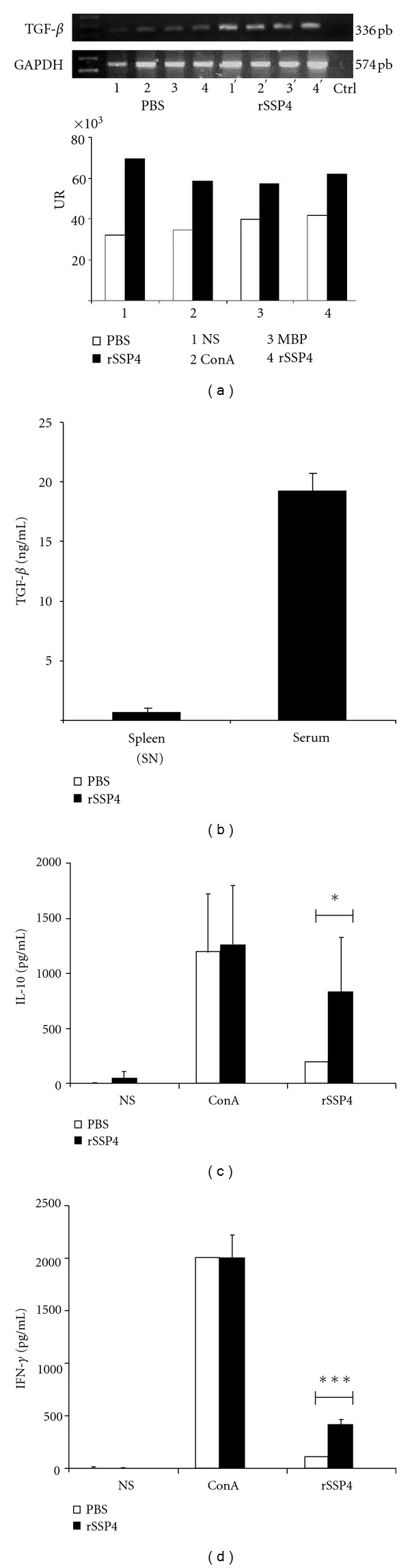
Immunization with rSSP4 induces TGF-*β* mRNA expression and TGF-*β*, IL-10, and IFN-*γ* production. RNA from 24 h cultured spleen cells was extracted and used to determine TGF-*β* expression by RT-PCR, and culture supernatants were harvested and assessed for cytokines production by ELISA (*n* = 3). (a) TGF-*β* mRNA expression by wild type spleen cells (upper panel); densitometric analysis of TGF-*β* mRNA expression (lower panel). Culture supernatants were harvested after 72 h of culture and assessed for TGF-*β*, IL-10, and IFN-*γ* production by ELISA. (b) TGF-*β* production was measured in culture supernatants (SNs) of spleen cells from rSSP4 immunized mice under rSSP4 restimulation condition and in sera from immunized mice. Graphs show values in pg/mL (mean ± SD). (c) and (d) IL-10 and IFN-*γ* production, respectively, by wild type mice in immunized (full bars) and nonimmunized mice (open bars) under different conditions: NS (non-stimulated), ConA, or rSSP4. Results shown are the average of at least six independent experiments done in triplicate. Differences between groups are considered significant at *P* less than 0.05 and are represented by *P < 0.05, and ***P < 0.001 in a Student *t*-test.

TGF-*β* and IL-10 have been implicated in the pathogenesis of *T. cruzi *infection, and high levels of both cytokines are usually associated with regulatory T cell differentiation [15, 16]. Once we observed the suppressive properties of Treg cells both *in vivo* and *in vitro*, we continue examining the role of rSSP4 on the immune response and continue to look for the presence of different cytokines, responsible of Treg cells differentiation and of their functions. High levels of TGF-*β* mRNA expression were found in cultured cells and the presence of the protein both in serum of rSSP4 immunized mice, and in culture supernatant of restimulated cells from immunized animals. A high level of TGF-*β* mRNA expression was found in immunized mice in comparison with mice that were not immunized, independently of the stimulation condition, suggesting that this immunomodulatory cytokine is induced by the amastigote-specific antigen SSP4 *in vivo* (Figure 4(a)). Furthermore, sera from rSSP4-immunized mice contained significantly higher levels of TGF-*β* compared to culture supernatant from rSSP4-stimulated T cells (Figure 4(b)). Because none of the other restimulation conditions induced TGF-*β*, these results show that rSSP4 is a potent inducer of this immunosuppressive cytokine. Proinflammatory cytokines such as IFN-*γ* contribute to host resistance against *T. cruzi,* whereas anti-inflammatory cytokine IL-10 has been implicated in mediating susceptibility; therefore, we examined the effect of rSSP4 on Th1/Th2 cytokines production by spleen cells. rSSP4-stimulated spleen cells from rSSP4-immunized mice produced significantly more IL-10 than similarly stimulated spleen cells from nonimmunized control mice. This difference between immunized and nonimmunized mice was not found upon mitogenic stimulation with ConA (Figure 4(c)). IL-10 production was also measured in cell culture supernatant from cells grown in the presence of polymyxin B; no differences in IL-10 production between presence and absence of polymyxin B were found indicating that LPS was not responsible for the increase in IL-10 production (data no shown). These data support previous observations where rSSP4 immunization increased levels of IL-10 mRNA expression in BALB/c mice [11].

With respect to IFN-*γ*, a key Th1 cytokine, differences were also observed under the assay conditions. As in the case of IL-10, there were no differences between the two groups when cells were stimulated with ConA. However, this cytokine was found in greater amounts in supernatant of spleen cells from immunized mice, restimulated with rSSP4 (Figure 4(d)).

## 4. Discussion

The results presented in this study indicate that CD4^+^ CD25^+^ regulatory T cells, induced by rSSP4, suppress cardiac pathology and prolong host survival during acute *T. cruzi *infection in a specific way. Still they contribute to disease progression by promoting peripheral blood parasitemia and cardiac parasite growth. In this study, we showed that rSSP4-primed CD4^+^CD25^+^ regulatory T cells play a decisive immunoregulatory function by decreasing inflammation and increasing survival and parasitemia in immunized and rSSP4-primed CD4^+^CD25^+^ T cells-transferred mice. Based on data from suppression assays, most probably, Treg cells exert their suppressive activity over CD4^+^ T cells in a partial TGF-**β** dependent mechanism. Even though immunized mice and transferred mice showed higher cardiac and blood parasitemia, they also showed better survival and reduced cardiac inflammation; these effects were not observed in nonimmunized mice or in mice transferred with naïve mice-derived CD4^+^CD25^+^ T. In these two groups, the same level of parasitemia and the same level of cardiac inflammation were observed. These results support our hypothesis that regulatory T cells induced by rSSP4 are antigen-specific.

Furthermore, results clearly show that *T. cruzi *amastigote stage-specific antigen SSP4 induces high levels of TGF-*β* and expansion of CD4^+^CD25^+^FOXP3^+^ T cells. These cells mediate suppression of effector T cells, via a TGF-*β*-dependent but IL-10 independent pathway. These results suggest that the high levels of IL-10 produced after rSSP4 immunization could be crucial for the differentiation process of CD4^+^CD25^+^FOXP3^+^ cells, but not for their suppressive mechanism. It is important to mention that rSSP4 induced an antigen-specific immune response, based on the fact that ConA, MPB, or absence of stimuli do not induce this population. Moreover, the induction of Treg cells does not occur by immunization with other *T. cruzi*-derived recombinant antigens [17]. 

To survive an infection requires that the host generates a controlled immune response that recognizes and eliminates the invading pathogen, while limiting collateral damage to self-tissues that may result from a vigorous immune response [18]. At the time of their first encounter with their host, parasites might modulate the immune response by actively secreting or expressing molecules with potent effects on the immune system [8, 18]. A large variety of modulatory parasite-derived proteins, lipids, and glycoconjugates has been described [10]. In the *T. cruzi* protozoan parasite, specifically in the amastigote stage, a surface glycoprotein named SSP4 was described by Andrews et al. [9]. This study found that newly transformed amastigotes, both intracellular and extracellular, express SSP4 that is bound to the plasma membrane by a GPI anchor. We had previously reported that SSP4 induces mRNA expression of pro- and anti-inflammatory cytokines from macrophages *in vitro *[11]. In the present study, we have extended the findings on the immunomodulatory function of *T. cruzi-*derived SSP4 showing that rSSP4 can induce the expansion of regulatory T cells during *T. cruzi *infection, accompanied by TGF-*β*, IL-10, and IFN-*γ* production.

Several pathogens have been reported to induce the expansion of Treg cell populations [16], including naturally occurring FOXP3^+^ Treg and induced Treg cells, including Tr1 [19] and Th3 cells [15]. Although CD4^+^CD25^+^ T cells have been identified as critical regulators of immune response during infections caused by different protozoa [20], their role in regulating the outcome of *T. cruzi *infection is not clear. A previous research by Kotner and Tarleton reported that depletion of regulatory T cells prior to *T. cruzi *challenge had no effect on the outcome of acute *T. cruzi *infection caused by a Brazilian strain [1]. In contrast, Mariano et al. (2008) found that CD4^+^CD25^+^GITR^+^FOXP3^+^ T cells migrate to the heart after *T. cruzi *challenge and that the administration of anti-CD25 or anti-GITR Ab resulted in increased mortality during infection [21]. In addition, this study found that anti-GITR treatment was associated with increased TNF-*α* production and myocarditis as well as tissue parasitemia. In this work, we found that *in vivo* exposure of passively transferred or immunized animals to SSP4 antigen, expressed by the amastigote stage, or *in vitro* by restimulation with rSSP4, induces the conversion of Treg cells CD25^+^FOXP3^+^. These cells were able to inhibit proliferation of effector CD4^+^ T cells *in vitro* and to promote peripheral blood and heart parasitemia *in vivo*. Sun et al. (2012) reported the induction of CD4^+^CD25^+^FOXP3^+^ Treg cells by rSj16, a recombinant protein derived from a protein present in the secretions of *Schistosoma japonicum* [22].

The role of IL-10 as an immunoregulatory cytokine in infection has been documented primarily in the context of chronic infections. IL-10 can suppress immune responses (either Th1 or Th2 cells) towards many pathogens in experimental models. The four major T-cell sources of IL-10 are T-helper type 2 (Th2) cells, subsets of regulatory T cells designated Tr1, Th1, and Th17 cells [23]. Nevertheless, cells such as macrophages, B cells, NK cells, and CD8^+^ T cells, which are involved in determining the outcome of *T. cruzi* infection, also produce IL-10. We examined different cell types, such as B cells, MΦs, NK, and T cells, as potential sources of rSSP4-induced IL-10. We found that at least for the rSSP4-antigen and under restimulation conditions, IL-10 is produced by CD4^+^CD25^+^ cells (data not shown). This is perhaps not surprising, since a recent study from our laboratory found that rSSP4 induces a population of IL-10/IFN-*γ* CD4^+^ double producers T cells [24], which have also been identified as a major source of IL-10 during infections such as leishmaniasis [25].

Taken together, these findings suggest that regulatory CD4+ T cells are involved in progression and pathogenesis of experimental *T. cruzi *infection. Indeed, clinical studies by Vitelli-Avelar et al. (2008), Fiuza et al. (2009), and de Araújo et al. (2011, 2012) found that individuals in the indeterminate clinical form of the disease have a higher frequency of CD4^+^CD25^high^ T cells population secreting IL-10 and expressing FOXP3, indicating that the balance between regulatory and effector T cells might be a critical determinant of disease progression in Chagas disease [26–29].

## 5. Conclusion

In conclusion, *T. cruzi* amastigote stage-specific protein SSP4 enhances production of TFG-*β* and IL-10 and induces expansion of CD4^+^ regulatory T cells in susceptible BALB/c mice. These cells suppress proliferation of effector CD4^+^ cells by an IL-10 independent mechanism but a partially TGF-*β*-dependent mechanism. In addition, CD4^+^CD25^+^ regulatory T cells from rSSP4 treated mice suppress cardiac inflammation and prolong survival, but promote peripheral blood and heart parasitemia when transferred into syngenic recipients just prior to *T. cruzi *challenge. These findings suggest that *T. cruzi *amastigote stage-specific protein SSP4 could contribute to immune evasion and establishment of chronic infection, by inducing expansion of disease exacerbating CD4^+^C25^+^ regulatory T cells during acute *T. cruzi *infection.

## Abbreviations

rSSP4: amastigote stage-specific recombinant protein;
MΦs: macrophages.
